# Trajectory of hearing loss in children with unilateral hearing loss

**DOI:** 10.3389/fped.2023.1149477

**Published:** 2023-04-11

**Authors:** Elizabeth M. Fitzpatrick, Flora Nassrallah, Isabelle Gaboury, JoAnne Whittingham, Bénédicte Vos, Doug Coyle, Andrée Durieux-Smith, Marie Pigeon, Janet Olds

**Affiliations:** ^1^ Faculty of Health Sciences, University of Ottawa, Ottawa, ON, Canada; ^2^ Child Hearing Laboratory, CHEO Research Institute, Ottawa, ON, Canada; ^3^ Faculty of Medicine and Health Sciences, Université de Sherbrooke, Longueuil, QC, Canada; ^4^ Audiology Clinic, CHEO, Ottawa, ON, Canada; ^5^ School of Public Health, Université libre de Bruxelles (ULB), Brussells, Belgium; ^6^ School of Epidemiology, Public Health and Preventive Medicine, University of Ottawa, Ottawa, ON, Canada

**Keywords:** unilateral hearing loss, children, progressive loss, trajectory, etiology

## Abstract

**Introduction:**

The aim of this study was to quantify the amount of deterioration in hearing and to document the trajectory of hearing loss in early identified children with unilateral hearing loss (UHL). We also examined whether clinical characteristics were associated with the likelihood of having progressive hearing loss.

**Methods:**

As part of the Mild and Unilateral Hearing Loss Study, we followed a population-based cohort of 177 children diagnosed with UHL from 2003 to 2018. We applied linear mixed models to examine hearing trends over time including the average amount of change in hearing. Logistic regression models were used to examine the relationship between age and severity at diagnosis, etiology, and the likelihood of progressive loss and amount of deterioration in hearing.

**Results:**

The median age of the children at diagnosis was 4.1 months (IQR 2.1, 53.9) and follow-up time was 58.9 months (35.6, 92.0). Average hearing loss in the impaired ear was 58.8 dB HL (SD 28.5). Over the 16-year period, 47.5% (84/177) of children showed deterioration in hearing in one or both ears from their initial diagnostic assessment to most recent assessment including 21 (11.9%) who developed bilateral hearing loss. Average deterioration in the impaired ear ranged from 27 to 31 dB with little variation across frequencies. Deterioration resulted in a change in category of severity for 67.5% (52/77) of the children. Analysis for children who were followed for at least 8 years showed that most lost a significant amount of hearing rapidly in the first 4 years, with the decrease stabilizing and showing a plateau in the last 4 years. Age and severity at diagnosis were not significantly associated with progressive/stable loss after adjusting for time since diagnosis. Etiologic factors (ENT external/middle ear anomalies, inner ear anomalies, syndromic hearing loss, hereditary/genetic) were found to be positively associated with stable hearing loss.

**Conclusion:**

Almost half of children with UHL are at risk for deterioration in hearing in one or both ears. Most deterioration occurs within the first 4 years following diagnosis. Most children did not experience sudden “large” drops in hearing but more gradual decrease over time. These results suggest that careful monitoring of UHL especially in the early years is important to ensure optimal benefit from early hearing loss detection.

## Introduction

Unilateral hearing loss (UHL) in children has gained increasing attention as a clinically important hearing disorder. Permanent childhood hearing loss is relatively common affecting 3–4 per 1,000 children when all degrees of bilateral and unilateral loss are considered during childhood (1). An estimated 20%–30% of these children have unilateral hearing loss (1, 2). In contrast to historical practices, a substantial proportion of children with UHL are now diagnosed in infancy or early childhood due to widespread population level newborn hearing screening (NHS) (3, 4). Permanent UHL affects about 1 per 1,000 infants based on newborn screening cohorts (5, 6).

Historically, the clinical implications of UHL were not well understood. Unlike children with bilateral hearing loss, these children have access to speech and develop spoken language without intervention. However, there is a growing consensus that UHL affects typical development of auditory pathways and auditory function with implications for communication and academic development for at least some children (7–10). Difficulties in language and academic performance can persist at school age (11–13). However, some uncertainty remains about the consequences of UHL and who is most at risk for difficulties and the need for intervention and overall best practices continue to receive attention (14–17). Parental uncertainty about the effects of UHL has been reflected in parent-focused literature and studies suggest considerable indecision about intervention recommendations (18–20).

There is some variation in NHS programs worldwide in defining hearing disorders including whether mild bilateral and UHL, historically considered to be minimal losses, are specifically targeted (17, 21, 22). Arguably, one reason for including UHL is that as a public health intervention, screening aims not only to improve developmental outcomes but also to prevent delay through early audiologic management and intervention. Programs such as the Infant Hearing Program in Ontario, Canada (23), target the early detection of UHL on the basis that there may be negative consequences associated with any hearing loss and that children are at risk for deterioration in hearing in the other ear. Several studies have reported that children with hearing loss are at risk for further deterioration in hearing with wide variation in rates of progressive loss documented (24–28). Purcell et al. (29) reported that 32.8% of 128 children with sensorineural UHL who had their first audiologic assessment at age 7.7 years showed progressive hearing loss. Paul et al. (30) reported that 19% of 80 children showed progressive loss but 68% of children were initially identified with severe-profound hearing loss and further deterioration in hearing thresholds may not have been captured. Importantly, there has been little focus on the trajectory of hearing loss in children with UHL (31), particularly in early identified children. Datasets available prior to NHS included few children with early-detected UHL, limiting the possibility to document changes in hearing (3). Therefore, little is known about when and how much change in hearing occurs.

Relatively little is known about the relationship between the clinical characteristics (e.g., etiology, age at diagnosis, severity of hearing loss) of children with UHL and the risk of progression in hearing loss. Like bilateral hearing loss, etiology is related to both genetic and environmental factors. While genetics are the most common cause of bilateral hearing loss (1, 32, 33), structural and environmental causes make up a large part of the etiological distribution of UHL (33, 34). While several environmental factors including prematurity and ototoxicity have been associated with non-genetic hearing loss, congenital cytomegalovirus (cCMV) has emerged as the most common cause (32, 35). Congenital CMV accounts for 15%–20% of childhood hearing loss, including UHL, and has been associated with both late onset and progressive hearing loss (36–39). However, in an etiologic study, Dahl et al. (40) found no relationship between CMV or common genetic etiologic factors and progression of hearing loss. Structural anomalies such as enlarged vestibular aqueduct (EVA) and cochlear nerve deficiency (29, 33), common causes of UHL, have also been associated with progressive loss (29, 41). In an investigation of children with UHL, Purcell et al. (29) reported that children with bony cochlear nerve stenosis were at greater risk of progression in hearing. In the same study, risk of progression was not significantly different for children with and without EVA or for those with temporal bone anomalies versus normal imaging results. Overall, the research suggests that the relationship between etiologic factors and the risk of progressive hearing loss is rather inconclusive.

The Joint Committee on Infant Hearing (JCIH) has historically identified risk indicators for late onset and progressive permanent hearing loss, which have guided screening surveillance programs (22, 42). Our previous research on a cohort of children with bilateral/unilateral loss found no significant association between risk indicators and progressive loss except that children with craniofacial anomalies were more likely to have stable hearing loss (24). Permanent conductive (structural) loss, generally associated with craniofacial anomalies such as aural atresia has been reported in 25%–33% of children with UHL (2, 43). Onset of hearing loss, which can be related to etiology, and type and severity of hearing loss at diagnosis have also not been well-investigated in relation to progressive hearing loss.

Understanding the trajectory of hearing loss has implications for management practices including the need for surveillance and potential adjustments in intervention. Screening aims to improve developmental outcomes by detecting and managing hearing loss early and provides new opportunities to better understand the evolution of childhood hearing loss. Consistent with these goals, we have followed a population-based cohort of children with permanent hearing loss in one Canadian audiology center. The purpose of this study was to examine the clinical characteristics and the evolution of hearing loss in children with UHL. Specifically, the objectives were to: (1) determine the proportion of children with UHL, the amount of deterioration in hearing thresholds, and the trajectory of hearing loss; and (2) examine whether there was an association between clinical characteristics at diagnosis including etiology, age at diagnosis (related to onset), and severity of hearing loss and the likelihood of progressive hearing loss.

## Methods

### Design and setting

This longitudinal study was conducted as part of a research program examining development outcomes in children with mild bilateral or UHL. As part of this project, population-level data related to diagnosis and intervention were collected prospectively on all children with permanent hearing loss followed in the Eastern Ontario region of Canada and diagnosed from 2003 to 2018. For this study on progressive hearing loss, we also extracted all post-diagnostic audiometric data from the medical records.

The study was conducted at CHEO, a pediatric hospital which is the sole audiologic diagnostic center for infants in the area screened through a province-wide early hearing detection and intervention (EHDI) program. Screening targets include mild bilateral and UHL. The clinic also provides services for children who relocate to the area. Well-established clinical protocols for identification and follow-up of hearing loss are in place (44). Services are publicly funded through the provincial health system. The program was fully implemented in 2003 and data for this study are population-based, covering a birth cohort of approximately 240,000 infants during the 16-year study period. Services for all children confirmed with permanent hearing loss include audiologic follow-up at 3- and 6-month intervals respectively in the first and second year after identification and then annually up to age 6 years. Intervention services for communication development are also provided within the audiology service.

### Participants

The study population included all children followed at CHEO who were identified with permanent UHL (2003–2018). UHL was determined based on the National Workshop on Mild and Unilateral Hearing Loss (45) definition as hearing loss in one ear only with a pure-tone average (PTA at 0.5, 1, 2 kHz) of 20 dB HL or >25 dB at two or more frequencies above 2 kHz. Research Ethics Committees at the CHEO Research Institute (file #09-64X), and the University of Ottawa (file #H10-09-11) approved the study protocol.

### Procedures

Data collection for this study took place in two phases. In phase 1, as part of a longitudinal study on all children with hearing loss, clinical characteristics have been collected prospectively from medical records since full NHS implementation in 2003. Data were entered in a study-specific database and included child (e.g., sex, screening status) and hearing loss details (e.g., onset, age of diagnosis, type of loss, severity of hearing loss, middle ear status, etiology, risk indicators). In addition, medical records were re-examined for this study to update the child's profile with any new etiologic information from clinical areas such as genetics (e.g., family history and/or genetic testing), infectious diseases (e.g., cCMV infections) and ENT services (e.g., imaging results). A researcher with experience in medical chart data also entered risk indicators for hearing loss based on JCIH (42) descriptions and coding was verified with an audiologist or physician if needed.

In addition to the audiologic data entered at diagnosis, all follow-up audiologic and hearing-related medical assessment results were retrospectively extracted from paper or electronic (after 2013) medical charts and entered into an SPSS database including audiometric thresholds and middle ear status (e.g., immittance results and ENT clinical notes). Category of hearing loss (mild, moderate, moderately severe, severe, profound) was assigned based on 4-frequency 0.5, 1, 2, 4 kHz) pure-tone average (PTA) applying standard audiologic definitions (shown in Table 1). All clinical assessment data were available to the researchers.

**Table 1:** Baseline clinical characteristics of the study sample (*n* = 177).

Characteristic	Study sample	Stable HL	Progressive HL
	*n* = 177	*n* = 93	*n* = 84^a^
**Sex, *n* (%)**			
Female	87 (49.2)	42 (45.2)	45 (53.6)
Male	90 (50.8)	51 (54.8)	39 (46.4)
**Screening status**			
Exposed to screening	134 (75.7)	69 (74.2)	65 (77.4)
Not exposed to screening	43 (24.3)	24 (25.8)	19 (22.6)
**Onset hearing loss, *n* (%)**			
Congenital/Early^b^	95 (53.7)	44 (47.3)	51 (60.7)
Late onset^c^	46 (26.0)	30 (32.3)	16 (19.0)
Acquired	7 (4.0)	6 (6.5)	1 (1.2)
Unknown	29 (16.4)	13 (14.0)	16 (19.0
**Age diagnosis (months), median (IQR)**	4.1 (2.1, 53.9)	24.3 (2.0, 58.9)	3.7 (2.2, 44.8)
Type of hearing loss, *n* (%)			
Sensorineural	119 (67.2)	57 (61.3)	62 (73.8)
Mixed	23 (13.0)	10 (10.8)	13 (15.5)
Conductive^d^	35 (19.8)	26 (28.0)	9 (10.7)
**PTA (4 frequency) at diagnosis (impaired/worse ear), mean (SD)**	58.8 (28.5)	63.3 (30.3)	53.8 (30.3)
**Degree of hearing loss at diagnosis (impaired/worse ear), *n* (%)**			
High frequency^e^	17 (9.6)	9 (9.7)	8 (9.5)
Mild (20–40 dB HL)	39 (22.0)	15 (16.1)	24 (28.6)
Moderate (41–55 dB HL)	33 (18.6)	18 (19.4)	15 (17.9)
Moderately severe (56–70 dB HL)	42 (23.7)	25 (26.9)	17 (20.2)
Severe (71–90 dB HL)	23 (13.0)	9 (9.7)	14 (16.7)
Profound (>90 dB HL)	23 (13.0)	17 (18.3)	6 (7.1)
**Risk factors at diagnosis, *n* (%)**			
Craniofacial anomalies	32 (18.1)	22 (23.7)	10 (11.9)
Syndromes (associated with HL)	9 (5.1)	3 (3.2)	6 (7.1)
Family history	9 (5.1)	4 (4.3)	5 (6.0)
NICU	7 (4.0)	1 (1.1)	6 (7.1)
CMV	4 (2.3)	0	4 (4.8)
Meningitis	4 (2.3)	3 (3.2)	1 (1.2)
Oncology treatment	3 (1.7)	3 (3.2)	0
No risk factors	109 (61.6)	57 (61.3)	52 (61.9)
**Etiology, *n* (%)**			
ENT anomaly-external/middle ear	28 (15.8)	22 (23.7)	6 (7.1)
ENT anomaly-inner ear	14 (7.9)	8 (8.6)	6 (7.1)
Syndrome (associated with HL)	21 (11.9)	8 (8.6)	13 (15.5)
Hereditary/genetic	15 (8.5)	8 (8.6)	7 (8.3)
CMV	8 (4.5)	1 (1.1)	7 (8.3)
NICU admission^f^	5 (2.8)	1 (1.1)	4 (4.8)
Meningitis	4 (2.3)	3 (3.2)	1 (1.2)
Oncology	3 (1.7)	3 (3.2)	0
Unknown	79 (44.6)	39 (41.9)	40 (47.6)
Total assessments, median (IQR)	7.0 (5.0, 11.0)	6.0 (5.0, 9.0)	9.0 (6.0, 14.0)
Time to most recent audiogram (months), median (IQR)	58.9 (35.6, 92.0)	50.6 (32.6, 88.5)	64.3 (39.3, 92.2)
Age at most recent audiogram (months), median (IQR)	87.5 (55.1, 139.0)	82.2 (52.1, 139.0)	88.8 (55.9, 140.6)

CMV, cytomegalovirus; ENT, ear, nose, and throat; HL, hearing loss; NICU, neonatal intensive care unit.

^a^ Includes 7 children who developed hearing loss in the normal hearing ear; impaired ear remained stable.

^b^ Early onset ≤6 months of age.

^c^ Late onset: >6 months of age.

^d^ Includes only permanent conductive hearing loss.

^e^ Defined as >25 dB HL at >2 frequencies above 2 kHz.

^f^ The children with NICU admission had no other determined etiologies (e.g., syndrome) and had one of the JCIH treatments or conditions (ECMO, assisted ventilation, ototoxic medication, and hyperbilirubinemia requiring exchange transfusion). A total of 16 children were admitted to the NICU but other children were classified in specific etiologic categories, e.g., 5 children with syndromes and 6 children with other etiologies such as CMV and ENT anomaly/inner ear.

#### Determination of progressive hearing loss

A definition used in our previous research (24), adopted from Dahl et al. (40), was applied: (1) a decrease of 10 dB or greater at two or more adjacent frequencies between 0.5 and 4 kHz or a decrease in 15 dB at one octave frequency in the same frequency range. Children were categorized as having progressive hearing loss (vs. stable hearing) if there was worse hearing in the impaired ear or if the ear with normal hearing developed a loss. The presence of progressive hearing loss was determined based on a comparison of initial and most recent audiologic profiles. The initial confirmation of permanent hearing loss was based on the audiologic assessment conducted, either diagnostic auditory brainstem response (ABR) testing (using tone pip stimuli) or behavioral audiometry results. The relationship between behavioral and ABR thresholds has been well-documented and correction factors have been established to predict behavioral thresholds from ABR results (47–49). For the, ABR results, clinical audiologists had recorded the estimated behavioural thresholds (eHL) in the medical chart, applying correction factors used by the Ontario Infant Hearing Program (47) and these eHL thresholds were entered for all ABR tests. Most children, due to their age, were assessed using behavioral audiometry at their most recent assessment. Therefore, determination of progressive hearing loss for children who were initially diagnosed using ABR assessments, required a comparison of ABR (eHL thresholds) and behavioral thresholds.

Decision rules consistent with our previous research on progressive hearing loss (24) were applied. Inconclusive or incomplete results were not included for the analysis. If middle ear function was abnormal (based on tympanometry and/or ENT medical chart notes) at any assessment, audiograms with >10 dB changes in thresholds compared to previous/subsequent assessments were excluded. Assessments which included sound field results only were also excluded. Any unclear results were discussed between two researchers and reviewed with a clinical audiologist on the research team, as needed. For each audiological assessment entered, time from the confirmation of the hearing loss was calculated in months. For longitudinal analysis, the audiometric thresholds closest to and within 6 months of the year of follow-up (e.g., year 1, 2, 3, etc.) were selected (e.g., Year 2 encompassed thresholds obtained between 18 and 30 months).

### Data analysis

Statistical analyses were conducted using SPSS (version 26). Participant characteristics were summarized using descriptive statistics including means and standard deviations, medians and interquartile ranges, and frequency counts as appropriate. One outcome of interest was the proportion of children with progressive hearing loss. Differences in clinical characteristics (e.g., onset, type, severity of hearing loss, etiology, risk indicators) were compared for children with progressive and stable hearing levels using *t*-tests or Mann-Whitney *U* tests (as appropriate) for continuous variables and chi-square tests for categorical variables.

The amount of change in hearing loss across frequencies was calculated from first to most recent audiometric assessment. For the longitudinal analysis, the trajectory of hearing loss (for the impaired ear at initial diagnosis) was analyzed using mixed linear models (with the identity correlation matrix) that were fit with maximum-likelihood estimation techniques to evaluate the trajectory across individual frequencies (0.5–4 kHz). To control for intra-subject variability of trajectories, a random effect was added on the linear term of the model. Another random effect on the intercept was added to control for the variability between individual baseline thresholds. The time effect was modeled as linear, quadratic, and cubic factors to be able to detect a loss in hearing (linear effect) and a change in the rate of decrease over time (quadratic and cubic effects). Analyses were conducted with all available data without imputation, as estimation of parameters using the maximum-likelihood method is considered adequate to address missing data (50, 51).

Using logistic regression, we also evaluated the relationship between clinical characteristics (age at diagnosis, severity of hearing loss at diagnosis, etiology) and status of hearing loss (stable versus progressive). Four multivariable models were also fit to evaluate the relationship between these covariates and the amount of deterioration in hearing at individual audiometric frequencies from 0.5–4 kHz. All models were adjusted for time since diagnosis. Two-tailed tests were applied for all analyses with statistical significance set at *p* < 0.05.

## Results

### Study population and characteristics

**Figure 1:**
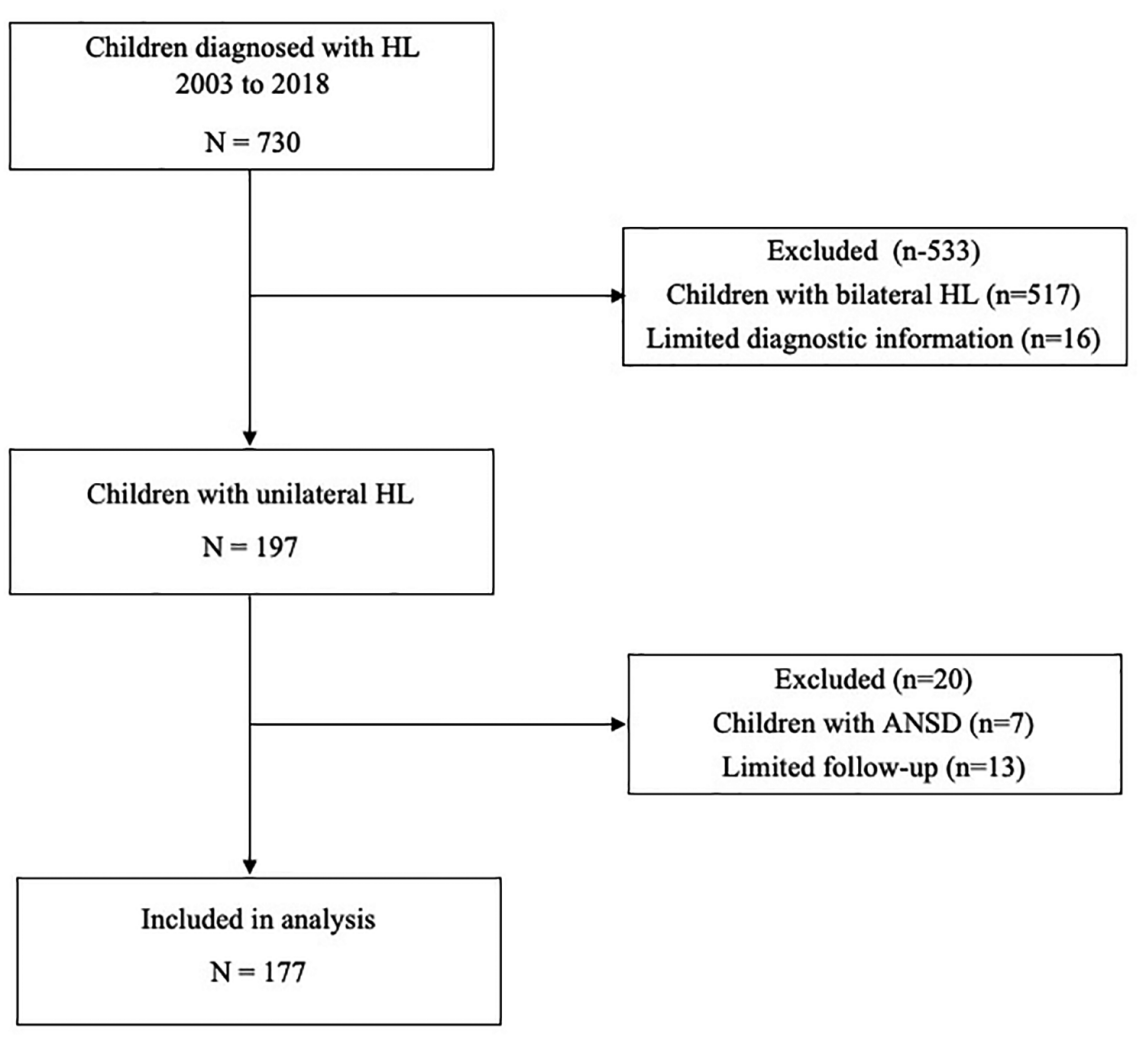
Selection of study participants. ANSD, auditory neuropathy spectrum disorder; HL, hearing loss.

Figure 1 shows the selection of participants for the analysis. From 2003 to 2018, a total of 730 children were identified with permanent hearing loss in the clinic, of whom 197 (27.0%) had UHL at diagnosis. After removing children with limited follow-up and those with auditory neuropathy spectrum disorder due to the fluctuating nature of hearing loss, 177 children were available for detailed analysis. A total of 1,565 audiologic assessments were examined (median of 7.0 assessments per child; IQR 5.0, 11.0; range 3–31) to determine whether hearing loss was progressive or stable.

### Description of participants

Table 1 shows the characteristics of the 177 children included in the analysis. Most children (134/177, 75.7%) were known to be exposed to newborn screening and UHL was diagnosed in infancy at a median age of 4.1 months (IQR 2.1, 53.9). Of the 43 (24.3%) children without screening, 17 (9.6%) were confirmed as not screened and 26 (14.7%) were born outside the province/country and were not screened or information was not available. Children had been followed for a median of 58.9 months (IQR 35.6, 92.0) and had a median age of 87.5 months (IQR 55.1, 139.0) at most recent assessment.

Hearing loss was determined to be congenital or early onset (<6 months) for 53.7% (95/177), late onset for 26.0% and acquired (e.g., meningitis or other known causes) for 4.0%. Onset was unknown for the remaining 16.4% of children due to unknown screening status and no early diagnostic assessment. Most (142/177, 80.2%) children presented with sensorineural (67.2%) or mixed (13.0%) hearing loss at diagnosis and the remaining 19.8% with permanent conductive (structural) loss. The mean hearing loss at diagnosis (4-frequency PTA in the impaired ear) was 58.8 dB (SD 28.5) with 74.0% (131/177) of children having <70 dB HL (mild to severe). One or more known risk indicators for hearing loss was documented for 38.4% (68/177) of the children. Etiology was known for 55.4% (98/177) of children with the most common etiologies being external/middle ear anomalies (15.8% of total), inner ear anomalies (7.9%), syndromes associated with hearing loss (11.9%) and hereditary/genetic causes (8.5%), together accounting for 88.7% of causes (details in Table 1).

### Proportion and severity of progressive loss

Overall, 84 of 177 (47.5%) children showed deterioration in hearing in one or both ears from initial diagnosis to most recent assessment. For 63 (35.6%) children, hearing loss remained unilateral with further deterioration in the impaired ear only, and 21 (11.9%) children developed bilateral hearing loss including 14 (7.9%) who showed deterioration in both ears since initial diagnosis and another 7 (4.0%) who developed a loss in the normal hearing ear only. For these 21 (11.9%) children, the loss in the normal hearing ear was identified at a median of 22.1 months (IQR 10.4, 43.3). In summary, 27.7% (98/354) of all ears showed a drop in hearing since initial diagnosis (77 impaired ears plus 21 previously unaffected contralateral ears).

Since this was an early identified cohort, we verified whether there was a difference in progressive hearing loss in children who were identified using objective ABR versus behavioral audiometry at baseline. At the final assessment there were 198 ears with hearing loss (177 impaired ears and 21 ears originally within normal limits), 51.5% (102 of 198) were identified through ABR testing at baseline and 48.5% with behavioral audiometry. Chi-square analysis showed no significant difference in the percentage of ears with progressive hearing loss when the initial diagnostic assessment was conducted using ABR versus behavioral audiometry [*X*^2^ (1) = 0.866, *p* = 0.352].

The characteristics of the 84 children with progressive hearing loss and the 93 with stable hearing are shown in Table 1. While there was no significant difference in the length of follow-up time for children with progressive loss compared to those with stable loss (*p* = 0.072), the children with progressive loss had more audiologic assessments (*p* < 0.001). As shown, more children with progressive hearing loss had congenital/early onset hearing loss (*p* = 0.042). Children with progressive hearing loss were diagnosed at a median age of 3.7 months (2.2, 44.8) compared to 24.3 months (2.0, 58.9) for those with stable hearing thresholds. It is important to note that children with late onset hearing loss are not necessarily identified at the initial onset of the loss but rather when it becomes severe enough to be noticed, therefore changes in hearing prior to diagnosis are unknown. There was no significant difference in age of diagnosis for children diagnosed with late onset hearing loss in the later 5-year period (2014–2018) compared to those diagnosed in the previous 11 years (2003–2013) (*p* = 0.074). Compared to children with stable hearing, children with progression had more sensorineural/mixed loss (*p* = 0.015) and had less severe hearing loss at diagnosis (*p* = 0.013). The latter finding may reflect that there were more children in the stable group with profound hearing loss at diagnosis in the impaired ear (17 vs. 6), and further deterioration may not have been captured if hearing loss had reached the limits of measured hearing thresholds. When considering only the 154 children with better than profound hearing loss, 50.6% (78/154) showed deterioration in at least one ear.

### Severity of hearing loss

**Figure 2:**
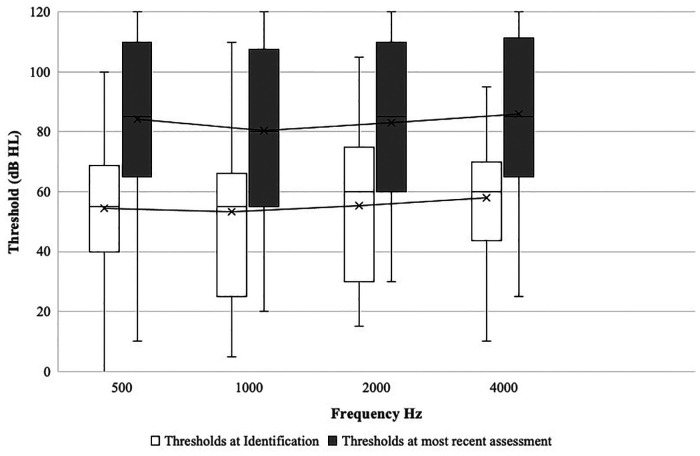
Average change in hearing thresholds across frequencies from initial diagnosis to most recent assessment (*n* = 77). The boxes indicate the 25th, 50th, and 75th percentiles. The whiskers above and below the box boundaries show the largest and smallest observed values. x on graph refers to mean thresholds.

Figure 2 shows the average drop in hearing by frequency (0.5–4 kHz) in the impaired ear for the 77 children with deterioration from first to last audiometric assessment. As shown, there was substantial deterioration in hearing across all frequencies. Average deterioration ranged from 27 to 31 dB with little variation across frequencies. For example, at diagnosis, average thresholds ranged from 53 dB HL at 1 kHz to 58 dB HL at 4 kHz and at last assessment from 80 to 86 dB HL. For the 21 children who developed bilateral loss (not shown in Figure 2), 16 (76.2%) children initially presented with high frequency only or mild hearing loss in the previously normal hearing ear; 14 showed further progression in that ear over time.

**Figure 3:**
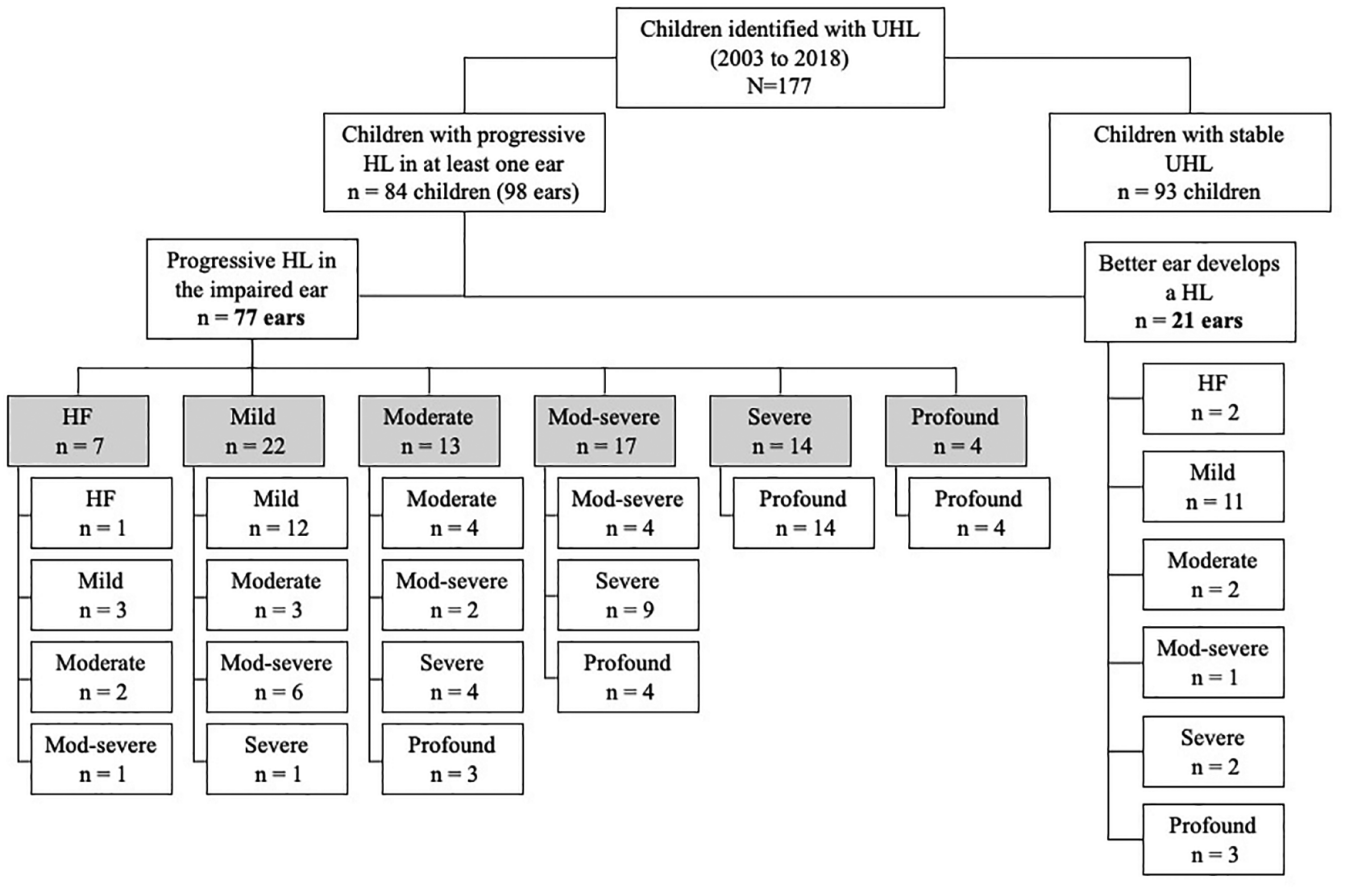
Category of hearing loss severity at diagnosis and at most recent assessment. The shaded boxes represent the degree of hearing loss in the impaired ear at diagnosis and the unshaded boxes the degree at final assessment. For the 21 ears that developed hearing loss (previously normal hearing), degree of hearing loss at final assessment is shown. UHL, unilateral hearing loss; HL, hearing loss.

Figure 3 shows the changes in category of severity of hearing loss for individual ears, classified according to PTA across the four frequencies (0.5, 1, 2, 4 kHz). Changes are shown separately for the 77 impaired ears and for the 21 ears that started with normal hearing. For the 77 impaired ears, deterioration was sufficient to result in a change in category of hearing loss severity for 67.5% (52/77). For example, 22 ears with mild hearing loss in the impaired ear at diagnosis showed a moderate or worse loss at last assessment and 14 moved from a severe to a profound loss category. For the 21 normal hearing ears, 8 showed a moderate hearing loss or greater at most recent assessment.

By definition, children with progressive loss did not show improvement in hearing levels. Of the 100 impaired ears that were coded as not progressive (stable), 3 showed >10 dB improvement (in 4-frequency PTA) from baseline to most recent assessment (range: 11.4–20.0 dB change). Two of these children had structural conductive hearing loss and all three children continued to present with hearing loss. One normal hearing ear that later developed hearing loss also showed an improvement of 13.8 dB from diagnosis to final audiogram but continued to show a mild loss.

### Trajectory of hearing loss

**Figure 4:**
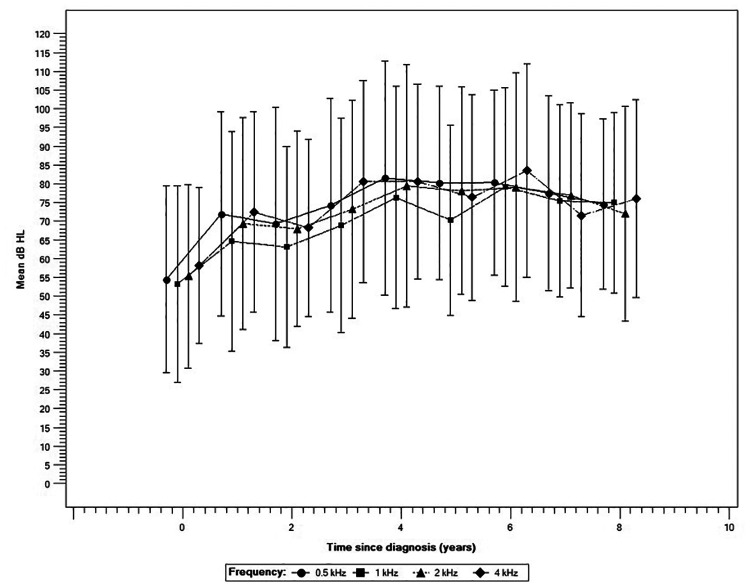
Trajectory of hearing loss across four frequencies (*n* = 77). Error bars represent one standard deviation.

Using the series of audiometric assessments recorded over the first 8 years of follow-up for this cohort (*n* at baseline of 48, 34, 60, and 50, for 0.5 1, 2 and 4 kHz respectively), we examined the trajectory of hearing loss in the impaired ear to document patterns of changes in hearing over time. Figure 4 shows that most children lost a significant amount of hearing rapidly in the first 4 years of follow-up (*p* < 0.001 for all four frequencies). On average, the loss was estimated at 27.1 dB, 23.1 dB, 24.1 dB, and 22.5 dB at 0.5 1, 2, and 4 kHz respectively. Subsequently, the decrease in thresholds showed a statistically significant stabilization in deterioration for all frequencies (*p* < 0.001) followed by a plateau in the last 4 years of observation.

### Relationship between child characteristics and progressive hearing loss

Logistic regression was carried out to assess the association between known clinical characteristics and progressive hearing loss in the 77 impaired ears that showed deterioration. Age at diagnosis and severity of hearing loss (4-frequency PTA) were not significantly associated with progressive/stable hearing loss (Table 2) after adjusting for time since diagnosis. However, the etiologic factors (ENT anomaly-external/middle ear, ENT anomaly-inner ear, syndrome, hereditary/genetic) entered in the model were found to be positively associated with stable hearing loss (i.e., protected against progressive hearing loss). For example, children with hereditary/genetic etiology have a 95% chance of stable hearing loss. However, given the relatively small number of children in some etiologic categories, the results should be interpreted with caution. After applying a Bonferroni correction [adjusted alpha level of 0.001—(.05/35)], only the factor ENT anomaly-external/middle ear remained statistically significant.

**Table 2:** Factors associated with progressive hearing loss (*n* = 77).

Factor	Adjusted odds ratio (95% CI)	*p*-value
Age at diagnosis	1.00 (0.98, 1.02)	0.733
Severity (PTA) at baseline	0.98 (0.96, 1.00)	0.103
**Etiologic factors**		
ENT anomaly-external/middle ear	0.01 (0.00, 0.13)	<0.001
ENT anomaly-inner ear	0.08 (0.01, 0.98)	0.049
Syndrome (associated with HL)	0.08 (0.01, 0.93)	0.044
Hereditary/genetic	0.05 (0.00, 0.71)	0.026
CMV^a^	n/a	n/a

CMV, cytomegalovirus; ENT, ear, nose, throat; HL, hearing loss; PTA, pure-tone average.

^a^ Insufficent number of participants for the regression model; 1 of 8 with CMV had stable hearing loss.

Using the same variables and adjusting for time since diagnosis, linear regression models were fit to examine the association between clinical characteristics and the total amount of deterioration in hearing at individual frequencies 0.5–4 kHz (Table 3). Age at diagnosis was not a significant predictor of change in hearing except at 0.5 kHz, where younger age at diagnosis was associated with more deterioration in hearing. This difference was small, translating to 2.2 dB more deterioration in the threshold at 0.5 kHz threshold when a child was diagnosed at age 12 months compared to 24 months. Applying a Bonferroni correction, (adjusted alpha level of 0.001) the result would no longer be statistically significant. There was no significant association between any etiology and amount of deterioration in hearing at any frequency.

**Table 3:** Factors associated with amount of deterioration in hearing across frequencies (*n* = 77).

	500 Hz		1,000 Hz		2,000 Hz		4,000 Hz	
	Coefficient^a^	*p*-value	Coefficient	*p*-value	Coefficient	*p*-value	Coefficient	*p*-value
Age at diagnosis (mos)	−0.18 (−0.35, −0.02)	0.028	−0.09 (−0.23, 0.06)	0.218	−0.01 (−0.21, 0.01)	0.095	−0.01 (−0.13, 0.11)	0.905
Severity (PTA) at baseline	0.01 (−0.26, 0.28)	0.935	0.10 (−0.13, 0.33)	0.383	−0.01 (−0.19, 0.16)	0.879	0.14 (−0.08, 0.35)	0.215
**Etiology factors**								
ENT anomaly-external/middle ear	−18.96 (−41.70, 3.78)	0.100	N/A		−14.18 (−46.90, 18.53)	0.388	−4.44 (−27.18, 18.31)	0.696
ENT anomaly-inner ear	0.47 (−24.80, 25.73)	0.971	−7.41 (−32.72, 17.90)	0.556	6.93 (−7.36, 21.22)	0.335	6.17 (−12.95, 25.29)	0.518
Syndrome (associated with HL)	−11.71 (−28.53, 5.11)	0.167	3.00 (−14.16, 20.16)	0.725	1.54 (−11.25, 14.32)	0.810	−2.25 (−16.71, 12.21)	0.755
Hereditary/genetic	8.25 (−33.12, 49.62)	0.689	4.30 (−14.12, 22.72)	0.638	0.62 (−13.66, 14.91)	0.930	−4.04 (−17.53, 9.45)	0.549
CMV	8.48 (−12.19, 29.15)	0.412	7.63 (−11.68, 26.93)	0.428	8.90 (−6.91, 24.71)	0.264	5.18 (−13.41, 23.77)	0.577

CMV, cytomegalovirus; ENT, ear, nose, throat; HL, hearing loss; PTA, pure-tone average.

^a^ In the table, coefficient refers to the difference in hearing level (at each frequency) for every 1 unit change in the factor examined (independent variable, e.g., age, severity). In the model, only age at diagnosis at 500 Hz was significant, i.e., for each month later age of diagnosis, there was a very slight improvement in hearing. For the etiologic (categorical) factors, the coefficient represents the amount of change in hearing level when the etiologic factor is not present vs. present. In the model, no coefficients were statistically significant, i.e., no factors were associated with a difference in hearing level at any frequency.

## Discussion

This population-based study showed that about 1 in 4 children with permanent hearing loss present with UHL at initial diagnosis. Based on a large dataset of longitudinal audiometric data, we found that almost half (47.5%, *n* = 84 of 177) of children first diagnosed with UHL experienced deterioration in hearing in the impaired ear or developed a hearing loss in the normal hearing ear. While deterioration for most children was limited to the impaired ear (43.5% of all children) 11.9% also developed bilateral hearing loss. We observed a trend towards a greater drop in hearing in the first 4 years after diagnosis with the decrease slowing over time and a plateau effect noted in the next 4 years.

Our overall findings related to the proportion of children who experience changes in hearing are consistent with our previous report on 330 children across the spectrum of hearing loss, both unilateral and bilateral loss (24). In that study 48% showed some amount of deterioration in hearing over time, including 37% of the 73 children with UHL. Almost half of all children had more than 20 dB drop in average hearing levels. In a subsequent study, we found that 42% of children with UHL showed deterioration, including 17% who developed bilateral loss (2). However, in both these studies we limited our analyses to a comparison of initial diagnostic and most recent audiologic results to determine progression. A study from another Canadian center reported that about one-third of 128 children with UHL showed progression (29). The current study adds another contribution to our understanding in detailing the trajectory of hearing loss. Through our analysis of multiple audiograms, we mapped out trends for children with UHL across a span of 8 years.

### Severity

The amount of change in hearing loss is important in planning optimal audiologic management of these children. During the study period, children lost an average of about 30 decibels across the individual speech frequencies (impaired ear) and more than two thirds of the deterioration happened over the first 4 years post-diagnosis (average of over 20 dB decrease in thresholds). These are clinically important changes. For example, for the 77 children with impaired ears that showed progressive hearing loss, the drop in average hearing levels was sufficient to result in two-thirds (67.4%) of them being reclassified to a more severe category of hearing loss at last audiometric assessment. This resulted in an almost doubling of the number of children with severe or profound hearing loss, in at least one ear (39 ears vs. 18 at diagnosis). Furthermore, 11.9% of children developed bilateral hearing loss placing them at greater risk for delays in auditory and communication development. Of the 21 normal hearing ears that developed a loss, 5 showed severe or profound hearing loss by study end.

The increase in severity of hearing loss in one or both ears is an important finding for two reasons. First, severity of hearing loss has important implications for the type of technology that these children are likely to require. Current guidelines generally support the use of conventional hearing aids for children with UHL who present with less than severe hearing loss (14, 16). For children with hearing aids, recommendations and counseling related to use may change with greater hearing loss severity. In addition, management options for children with severe to profound hearing loss, commonly referred to as single-sided deafness, have evolved in recent years with more children now considered for cochlear implants (15, 52–54). Changes in hearing might lead to different hearing technology (e.g., cochlear implant) and management options being considered for about one-quarter of the children (24.9%, *n* = 44) in our study compared to 10.2% at initial diagnosis. A recent review primarily based on adult UHL studies suggests that early cochlear implantation can prevent or reduce auditory deprivation in individuals with UHL (55). Secondly, there is some evidence from a systematic review to indicate that children with severe and profound UHL have more difficulty than those with less severe loss in at least some aspects of speech and language development (11). Earlier awareness of the presence of more severe loss may result in the fitting of optimal technology and provision of speech-language intervention in a timely manner, therefore, careful monitoring of these children would seem to be warranted.

### Trajectory

Knowing about the trajectory of hearing loss and any change in audiometric profiles over time can provide useful information for parents and can underscore the importance of monitoring their child's hearing. It can also be useful for clinicians and decision-makers in establishing appropriate clinical follow-up protocols. Our longitudinal analysis showed that the most important changes in hearing levels were observed in the first 4 years. Hearing continued to decrease over time but at a slower rate and the drop was much less pronounced 5–8 years after diagnosis. It is of clinical importance that most children did not experience sudden “large” drops in hearing but a more progressive, gradual decrease over time. These small changes in hearing thresholds are likely not noticeable by parents or therapists who see the children in everyday environments, especially since most continue to have one ear with normal hearing. When hearing loss drops suddenly, services may be initiated quickly. In contrast, our findings of more gradual progression indicate the need for close surveillance of hearing in these children in the first few years after diagnosis. Greater awareness about the possibility of worse hearing in one ear or the development of hearing loss in the contralateral ear can be valuable in guiding the families of these young children. For example, our previous research has shown that hearing aid use in the preschool years tends to be lower in these children, even when compared with mild bilateral loss (56). Timely information about a change in hearing may help parents decide to move forward with recommendations for amplification or motivate them to increase their child's hearing aid use. It is possible that concrete information about the evolution of UHL may influence parents’ decisions early in their child's life and potentially prevent or reduce delays in later childhood.

### Factors

Predicting who is most likely to lose further hearing would also be helpful in guiding families and in establishing clinical protocols. However, our examination of factors showed no clear relationship with age or severity of hearing loss at diagnosis. Our analysis of the available etiologic factors showed only that all were associated with stable hearing loss. In a previous study investigating risk factors (24), we also found a positive association between structural conductive conditions (e.g., atresia) and stable hearing loss. In that research, there was no relationship between any other risk factors and progressive/stable hearing loss. Dahl et al. (40) also did not find a relationship between severity of hearing loss or etiology and progressive loss over the first 3 years of life. It is important to note that our study was conducted prior to the implementation of cCMV or systematic genetic screening in the hearing program, resulting in almost half of the sample having unknown etiology. With further expansion of molecular screening in population-based NHS to detect infants at risk for late onset or progressive loss, more comprehensive analyses in sub-populations of children may eventually shed further light on progressive hearing loss (32, 57). In our study, we also could not show any relationship between age at diagnosis and the magnitude of deterioration in hearing levels across the speech frequencies except a small difference at 0.5 Hz, where a diagnosis 12 months earlier resulted in a loss of 2.2 dB more hearing per year. The etiologies examined also had no significant impact on the amount of deterioration in hearing.

### Limitations

A strength of this study is access to a population-based cohort in a publicly funded, health care system with comprehensive medical chart data available. Well-established diagnostic and follow-up protocols were in place in the clinic. Clinical characteristics and initial audiometric information were collected prospectively as children were diagnosed. However, our study has some limitations. A comparison of early audiologic (ABR and behavioral thresholds) and later behavioral assessments can introduce some error. Although we used estimated behavioral thresholds (eHL) to document ABR results, the agreement with behavioral thresholds is not perfect and there is some evidence that predicted behavioral thresholds may be underestimated in children with moderate and greater degrees of hearing loss (49, 58). In addition, behavioral threshold responses obtained for infants and young children, with normal hearing, particularly for VRA, are likely to be in the 20 dB HL range and become lower as their age increases (59, 60). This could lead to some underestimation of the number of children who experienced deterioration in hearing.

Our study depended on clinical audiologic data and despite clear follow-up protocols, the nature of clinical management is that assessments do not always follow the planned schedule. For young children, assessment results may be incomplete or require several test sessions. Furthermore, compared to children with bilateral hearing loss, audiologic follow-up for these children may be less consistent due to less concern on the part of parents about communication development, less frequent intervention sessions, and higher levels of amplification non-use. It is possible that children are less likely to present for follow-up visits if there is no concern. In addition, children in this study were diagnosed over a 16-year period and had variable lengths of follow-up. While we controlled for time since diagnosis in the regression analyses, this resulted in a smaller sample size for the longitudinal analysis of trajectory of hearing loss and requires that these results be interpreted with caution. Finally, the lack of specific etiologic data (e.g., based on radiologic findings or cCMV screening) and the relatively small number of children in some etiology groups precluded more extensive analyses of conditions (e.g., cCMV, enlarged vestibular aqueduct) previously reported to be associated with progressive hearing loss (37, 39, 41).

## Conclusion

Early identified children with UHL represent a new clinical population in the last 20–30 years since the widespread implementation of NHS. An important goal of screening is to improve developmental outcomes for children with hearing loss of any degree. Using population-level data to track the evolution of hearing loss, this study provides evidence that almost half of the children with UHL are at risk for further deterioration in hearing in the impaired ear or for bilateral loss especially in the first 4 years after diagnosis. The extent of the problem and the magnitude of the hearing deterioration, coupled with the potential impact on intervention decisions seem to justify efforts to regularly monitor these children to identify additional needs as early as possible.

## Data Availability

The datasets presented in this article are not readily available because Data are not available outside of the research team as per Ethics approvals. Requests to access the datasets should be directed towards the corresponding author.
